# Novel *CYP2A7*/*CYP2A6* germline hybrids associated with mutation burden in lung cancer revealed by whole-genome long-read sequencing

**DOI:** 10.1038/s41598-025-29542-w

**Published:** 2025-11-21

**Authors:** Sumiko Ohnami, Keiichi Hatakeyama, Takeshi Nagashima, Kouji Maruyama, Mitsuhiro Isaka, Fukumi Kamada, Sou Nakatani, Yuji Shimoda, Maki Mizuguchi, Akane Naruoka, Katsumasa Miyake, Keiichi Ohshima, Shumpei Ohnami, Yasuto Akiyama, Kenichi Urakami, Ken Yamaguchi

**Affiliations:** 1 Cancer Diagnostics Research Division, Shizuoka Cancer Center Research Institute, Nagaizumi-cho, Sunto-gun, Shizuoka, 411-8777 Japan; 2 Cancer Multiomics Division, Shizuoka Cancer Center Research Institute, Nagaizumi-cho, Sunto-gun, Shizuoka, 411-8777 Japan; 3 Experimental Animal Facility, Shizuoka Cancer Center Research Institute, Nagaizumi-cho, Sunto-gun, Shizuoka, 411-8777 Japan; 4 Division of Thoracic Surgery, Shizuoka Cancer Center Hospital, Nagaizumi-cho, Sunto-gun, Shizuoka, 411-8777 Japan; 5 Drug Discovery and Development Division, Shizuoka Cancer Center Research Institute, Nagaizumi-cho, Sunto-gun, Shizuoka, 411-8777 Japan; 6 Medical Genetics Division, Shizuoka Cancer Center Research Institute, Nagaizumi-cho, Sunto-gun, Shizuoka, 411-8777 Japan; 7 Immunotherapy Division, Shizuoka Cancer Center Research Institute, Nagaizumi-cho, Sunto-gun, Shizuoka, 411-8777 Japan; 8 Shizuoka Cancer Center, Nagaizumi-Cho, Sunto-gun, Shizuoka, 411-8777 Japan

**Keywords:** CYP2A6, Lung cancer, Structural variants, Long-read whole-genome sequencing, Tumor mutation burden, Genomic analysis, Next-generation sequencing, Sequence annotation

## Abstract

Nicotine is metabolized to cotinine by CYP2A6. *CYP2A6* genotypes are associated with variations in enzymatic activity and increase the risk of developing tobacco-related diseases, including lung cancer. The high sequence similarity between *CYP2A6* and *CYP2A7*, combined with frequent duplications, deletions, and structural variants (SVs), such as hybrids, complicate the accurate determination of *CYP2A7/CYP2A6* sequence. In this study, we investigated *CYP2A6* SVs in 20 Japanese patients with solid cancers using long-read whole-genome sequencing (lrWGS) and short-read whole-genome sequencing (srWGS). Combined lrWGS and srWGS facilitated the detection of copy number variations and SVs, including *CYP2A7/CYP2A6* hybrids. Three novel *CYP2A7/CYP2A6* hybrids were identified and validated via Sanger sequencing. Patients with *CYP2A6* deletion exhibited a lower tumor mutation burden (TMB) than those with normal *CYP2A6*. There was no increase in TMB in tumors other than lung cancer in smokers, even when germline *CYP2A6* was retained, indicating that the identified germline hybrids affect mutation accumulation in lung cancer in patients with a history of smoking. Overall, combined srWGS-lrWGS facilitated the precise determination of SVs between *CYP2A6* and *CYP2A7*. This approach could contribute to SV identification in highly homologous chromosomal regions and elucidation of functions of germline SVs in cytochrome P450 in lung cancer.

**Supplementary Information:**

The online version contains supplementary material available at 10.1038/s41598-025-29542-w.

## Introduction

The *CYP2A6* gene encodes an enzyme involved in the metabolism of nicotine and tobacco-specific carcinogens, and its genotype is associated with variations in enzymatic activity. Humans lacking *CYP2A6* exhibit reduced nicotine metabolism, which reduces their risk of developing tobacco-induced cancer^1,2^. Detection of *CYP2A6* variants may provide important insights into the development and prevention of smoking-related cancers, particularly lung cancer ^3–5^, although their clinical utility in cancer risk assessment remains unestablished^6^.

Recent advances in next-generation sequencing (NGS) have driven progress in pharmacogenomic research. NGS technologies facilitate a comprehensive assessment of common and rare genetic variants, enabling the determination of drug effectiveness and the risks of adverse drug reactions or therapeutic failure. Although short-read NGS can detect many structural variants, accurate resolution of gene conversions, hybrid alleles, and complete deletions remains challenging in highly homologous loci such as the cytochrome P450 (CYP) gene family (e.g., *CYP2A6/CYP2A7*)^7^.

We previously reported a WES-based single-assay that simultaneously profiled representative variants across multiple drug-metabolizing enzyme (DME) genes using blood from 2042 Japanese cancer patients^8^. Therein, we encountered difficulties in optimizing the identification of *CYP2A6* variants using our analytical platform^8^, similar to the challenges noted in the germline information published in the public Japanese Multi Omics Reference Panel jMorp (https://jmorp.megabank.tohoku.ac.jp/). In particular, the quality of single-nucleotide variant (SNV) information for *CYP2A6* is low in jMorp, likely because high sequence identity with the adjacent paralog *CYP2A7* and/or the frequent occurrence of hemizygous deletions leads to extensive filtering by variant quality score recalibration (VQSR).

To address this issue, we independently developed a genetic panel covering the exon regions of 20 DME genes, including *CYP2A6*^9^. This method combines long-range multiplex PCR with allele-specific primers and NGS, facilitating common *CYP2A6* variant analysis. In germline DNA, a comparison of genotypes between lung adenocarcinoma (LUAD) and lung squamous cell carcinoma (LUSC) using the DME panel revealed whole-gene deletion of *CYP2A6* (CYP2A6*4) in LUAD but not in LUSC. In that cohort, most CYP2A6*4 carriers were female non-smokers. Whole-gene deletion of *CYP2A6* is more frequent in East Asians (minor allele frequency [MAF] ~ 17% allele frequency) than in Western populations (e.g., MAF ~ 1% in Europeans and ~ 4% in admixed Americans)^10^. Taken together—the enrichment of germline *CYP2A6* deletion in LUAD (predominantly female non-smokers), its absence in smoking-related LUSC, and the higher Whole-gene deletion of *CYP2A6* frequency in East Asians—these observations motivate a testable hypothesis that germline disruption of *CYP2A6* may modulate tobacco-related mutational processes in lung cancer. However, accurate allele typing of the *CYP2A6* locus is challenging with our prior PCR–WES approach, particularly in the presence of copy-number variants, structural variants, and *CYP2A7/CYP2A6* hybrids. Short reads map ambiguously across the paralogs *CYP2A6* and *CYP2A7* genes and cannot delineate hybrid or gene-conversion breakpoints. Moreover, because WES targets only exons, coverage across intronic and intergenic regions is minimal, limiting the resolution of breakpoints and allele-level copy number.

The small number of accurately readable regions in highly homologous regions, such as *CYP2A6* and *CYP2A7*, is a major limitation of short-read whole-genome sequencing (srWGS; hereafter SRS). Long-read whole-genome sequencing (lrWGS; hereafter LRS) captures substantially longer-range information than SRS, facilitating the identification of regions with highly repetitive or duplicated sequences or regions with high homology^11,12^.

In this study, we combined LRS and SRS on the Illumina platform to improve the detection accuracy of *CYP2A6* alleles located in highly homologous regions and expand the identification of unknown SVs. Our ultimate goal was to enhance the current understanding of the biological role of smoking in lung carcinogenesis.

## Results

### Patient selection

Blood (not tumor) samples for germline analysis were obtained from 20 patients with solid cancers (11 LUAD, three LUSC, and two rectal adenocarcinoma [READ] cases, and one each of colon adenocarcinoma [COAD], esophageal squamous cell carcinoma [ESCC], clear cell renal cell carcinoma [CCRC], and gastrointestinal stromal tumor [GIST]) during surgery at the Shizuoka Cancer Center Hospital. The samples were subjected to Illumina LRS and SRS. In five patients with LUAD, we detected a germline *CYP2A6* whole deletion, previously detected via DME panel sequencing^9^. The remaining 15 cases were selected, including samples with intermittent CNVs detected previously using SRS (*n* = 13) and samples without any changes (*n* = 2) for use as controls^13,14^. The clinical and genomic characteristics of the selected patients are listed in Table 1.

**Table 1:** Patient characteristics.

Patient No	Sex	Age (years)	Smoking status^a^	Tumor type	*CYP2A6* CNV
1	Female	79	Never	LUAD	0.2
2	Male	86	Heavy	LUAD	0.0
3	Female	81	Heavy	LUAD	0.0
4	Male	73	Heavy	LUAD	0.0
5	Female	68	Heavy	LUAD	0.0
6	Female	78	Never	LUAD	1.1
7	Male	63	Heavy	LUAD	1.1
8 (ctl)	Female	58	Never	LUAD	2.0
9 (ctl)	Male	74	Heavy	LUAD	2.0
10	Male	75	Heavy	LUSC	3.0
11	Female	79	Heavy	LUSC	3.0
12	Male	81	Heavy	LUSC	2.9
13	Male	79	Heavy	LUAD	1.7
14	Male	75	Heavy	LUAD	1.7
15	Female	74	Never	READ	3.0
16	Male	71	Heavy	READ	2.9
17	Female	68	Never	COAD	1.9
18	Male	50	Heavy	GIST	1.9
19	Male	75	Light	ESCC	1.4
20	Male	48	Heavy	CCRCC	1.4

^a^ Pack-years was calculated by multiplying the number of packs of cigarettes smoked per day by the number of years of smoking and classified as follows: 0, never; 0–20, light; ≥ 20, heavy.

ctl, control; LUAD, lung adenocarcinoma; LUSC, lung squamous cell carcinoma; READ, rectal adenocarcinoma; COAD, colon adenocarcinoma; GIST, gastrointestinal stromal tumor; ESCC, esophageal squamous cell carcinoma; CCRCC, clear cell renal cell carcinoma.

### Combined SRS and LRS analysis

**Fig. 1:**
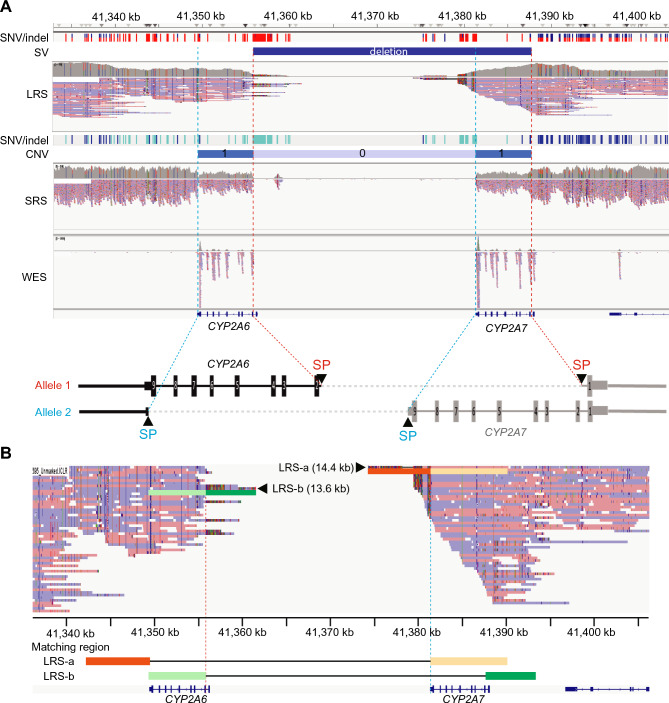
Integrative genomics viewer (IGV) images of a representative case (case No. 17) with a novel *CYP2A7/CYP2A6* hybrid allele. (**A**) The upper panel shows long-read sequencing (LRS), short-read sequencing (SRS), and whole-exome sequencing calls using IGV. Structural variant (SV) and copy number variant (CNV) regions detected via LRS and SRS are shown. The red and blue dotted lines represent the estimated switch positions (SPs). The lower panel shows the genomic structure and SPs of each allele determined using LRS and SRS. The grey dotted line represents deleted genome regions. (**B**) Visualization of the regions where LRS read sequences match the IGV image. LRS-a and LRS-b, containing a large number of mismatched sequences in a specific region, and the regions where these sequences are matched on the IGV image are color-coded for visualization, along with the locations of the *CYP2A7* and *CYP2A6* genes in the lower panel. LRS sequence lengths are indicated in parentheses.

The average whole-genome sequencing depth for the blood samples was 52.8 × for SRS and 81.1 × for LRS. The average sequence length of LRS was 4698 ± 2945 bp (mean ± standard deviation). SVs, CNVs, and SNVs, including indels, in the LRS and SRS data of the 20 samples were visualized using Integrative Genomics Viewer (IGV) (Supplementary Figure S1). SVs were detected in 18 samples; these included deletions in the *CYP2A7* and *CYP2A6* (transcribed on the reverse strand) regions. Figure 1A shows a representative case (case No. 17) with a novel *CYP2A7/CYP2A6* hybrid allele and *CYP2A6* deletion. In the SRS analysis, exons 2–9 in *CYP2A7* and *CYP2A6* showed a copy number of 1 and the intergenic regions of these genes showed a copy number of 0. Furthermore, LRS analysis revealed SVs, including large deletions from exon 1 of *CYP2A7* to exons 2–9 of *CYP2A6*. In the IGV image of LRS, sequence reads with high numbers of mutations (colored sequence reads in Fig. 1) at the end of reads were mapped to the region identified as CN0 via SRS. These reads covered more than 10 kb, and more than a third of the sequences in LRS-a and LRS-b (the longest upstream and downstream reads) could be mapped downstream of *CYP2A6* and upstream of *CYP2A7*, respectively (Fig. 1B). When these sequence reads (2–3 kb) were subjected to BLAT search (https://genome.ucsc.edu/cgi-bin/hgBlat), the downstream termination read of *CYP2A7* and upstream termination read of *CYP2A6* were identical to the *CYP2A6* and *CYP2A7* sequences, respectively. The detailed BLAT alignment results are provided in Supplementary Figure S2. This observation suggests that re-alignment with BLAT can improve the accuracy of LRS mapping in regions where standard alignments appear ambiguous. Similar applications of BLAT have been reported previously, for example in resolving paralogous gene structures^15^ and clarifying ambiguous SV alignments^16^. Taken together, these results indicated that each allele may have a different hybrid of *CYP2A7* and *CYP2A6*.

**Fig. 2:**
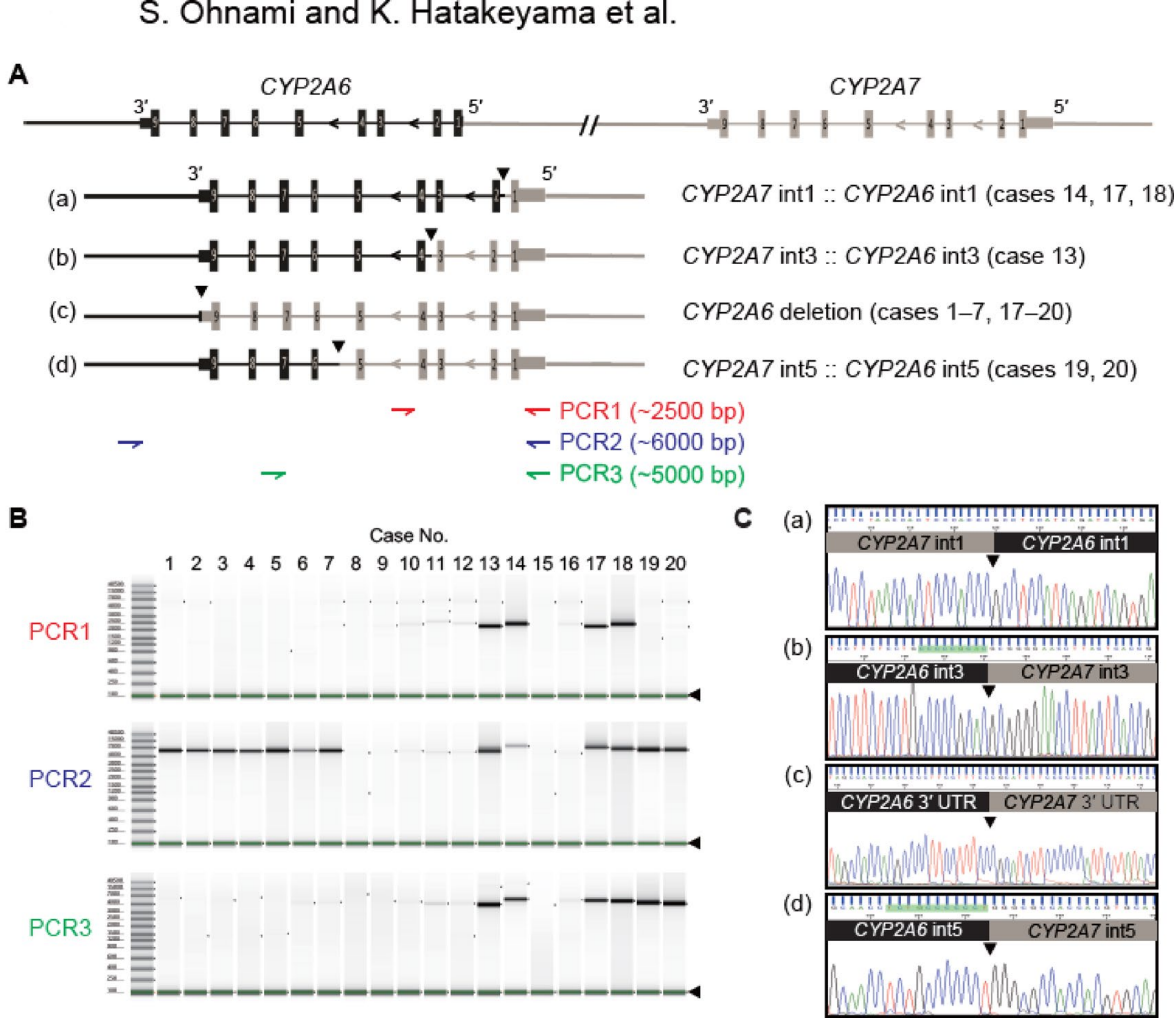
Validation of the identified *CYP2A7*/*CYP2A6* hybrid alleles. (**A**) Schematic representation of the genomic structure and breakpoints in four hybrids between *CYP2A6* and *CYP2A7*. The hybrids (a), (b), and (d) are not registered in the database (PharmVar). Hybrid (c) is a known complete deletion-type hybrid of the *CYP2A6* coding region. The triangle indicates the estimated switch position (SP). The positions of the PCR primers used for verification are shown below the hybrids. int, intron. (**B**) PCR amplicon length determined using TapeStation. The gel in the electrophoresis lanes, including the markers, was physically independent on the TapeStation and was automatically adjusted by the lower marker (black triangle), after which the electrophoresis results were output. The electrophoresis lanes were trimmed and sorted by case number. Raw data of original gels are presented in Supplementary Figure S4. (**C**) Verification of the sequence in the amplicon and estimation of SP. (a)–(d) correspond to hybrids (a)–(d) in Fig. 2A. UTR, untranslated region.

*CYP2A6* and adjacent *CYP2A7* alleles were PCR-amplified using three primer combinations to validate the SV data. The amplicons were subjected to Sanger sequencing to confirm the presence of *CYP2A7*/*CYP2A6* hybrid alleles. Figure 2 shows the three novel CYP2A7/CYP2A6 hybrids (a, b, d) and one known CYP2A6 deletion (c), which corresponds to the PharmVar-defined *4 allele, identified and verified in this study. The first type (a) was a hybrid allele, with switch position (SP) between *CYP2A7* intron 1 and *CYP2A6* intron 1, observed in three patients (case nos. 14, 17, and 18). The second (b) and third types (d) were hybrid alleles with an SP in *CYP2A7* intron 3 and *CYP2A6* intron 3 (case No. 13) and *CYP2A7* intron 5 and *CYP2A6* intron 5 (case Nos. 19 and 20), respectively. PCR primers were designed as a screening assay to detect the presence of a *CYP2A7::CYP2A6* junction within the targeted segment rather than to discriminate among junction positions by amplicon size. For example, in samples 13 and 17, the PCR2 and PCR3 bands differed in size according to the distinct junctions, and all junctions were then confirmed by Sanger sequencing. The known deletion type (c) was detected as a hybrid with an SP in the 3′ untranslated regions (UTRs) of *CYP2A7* and *CYP2A6*. All novel *CYP2A7*/*CYP2A6* hybrids identified using combined LRS and SRS were confirmed using PCR and Sanger sequencing.

Table 2 summarizes the estimated SP sequences in *CYP2A7/CYP2A6*. Five of the 20 patients showed homozygous deletions in *CYP2A6* (case Nos. 1–5) and five showed *CYP2A6* duplications (case Nos. 10–12, 15, and 16). *CYP2A6* heterozygous deletions were observed in two patients (case Nos. 6 and 7). In nine patients, an intact *CYP2A6* allele was not retained in the genome due to the presence of *CYP2A7*/*CYP2A6* hybrids or complete deletion of the coding region (“deletion” in Table 2). Hereafter, we use functional categories: ‘deletion type’ (functionally deficient) denotes biallelic loss of CYP2A6 function (e.g., CYP2A6*4 or two predicted no-function alleles including *CYP2A7/CYP2A6* hybrids), whereas ‘retained type’ (functionally non-deficient) denotes the presence of at least one intact *CYP2A6* allele*.* The structure and SPs of these hybrids and *CYP2A6* deletion types are shown in Supplementary Table S1. In this table, allele (c) is annotated as CYP2A6*4, whereas the novel hybrid alleles (a, b, d) do not correspond to previously reported star alleles (e.g., *12, *34, and *47). For reference, CYP2A6*12 is described as SV:CYP2A7::CYP2A6 hybrid (exons 1–2 from *CYP2A7*), CYP2A6*34 as SV:CYP2A7::CYP2A6 hybrid (exons 1–4 from *CYP2A7*), and CYP2A6*47 as SV:CYP2A7::CYP2A6 hybrid (exons 1–8 from *CYP2A7*). Following this format, our novel alleles are presented as novel (a): SV:CYP2A7::CYP2A6 hybrid (exon 1 from *CYP2A7*), novel (b): SV:CYP2A7::CYP2A6 hybrid (exons 1–3 from *CYP2A7*), and novel (d): SV:CYP2A7::CYP2A6 hybrid (exons 1–5 from *CYP2A7*). Notably, three patients had *CYP2A7*/*CYP2A6* hybrids (case Nos. 17–20) with a complete loss of functional domains, which may affect the metabolism of precancerous nicotine-related nitrosamines in cigarette smoke and other CYP2A6 substrates.

**Table 2:** Overview of SVs and alleles in the *CYP2A7* and *CYP2A6* regions.

Patient No	Tumor type	PCR amplicon^a^	SVs in *CYP2A7/CYP2A6* region (CYP2A7::CYP2A6)^b^		*CYP2A6* in genome^c^
			Allele 1	Allele 2	
1	LUAD	(c)	IGR::IGR	IGR::IGR	deletion
2	LUAD	(c)	3′ UTR::3′ UTR	IGR::IGR	deletion
3	LUAD	(c)	3′ UTR::3′ UTR	3′ UTR::3′ UTR	deletion
4	LUAD	(c)	3′ UTR::3′ UTR	3′ UTR::3′ UTR	deletion
5	LUAD	(c)	3′ UTR::3′ UTR	3′ UTR::3′ UTR	deletion
6	LUAD	(c)	3′ UTR::3′ UTR	Wild-type::Wild-type	retained
7	LUAD	(c)	3′ UTR::3′ UTR	Wild-type::Wild-type	retained
8	LUAD	N.A	Wild-type::Wild-type	Wild-type::Wild-type	retained
9	LUAD	N.A	Wild-type::Wild-type	Wild-type::Wild-type	retained
10	LUSC	N.A	Wild-type::Dup	Wild-type::Wild-type	retained
11	LUSC	N.A	Wild-type::Dup	Wild-type::Wild-type	retained
12	LUSC	N.A	Wild-type::Dup	Wild-type::Wild-type	retained
13	LUAD	(b)/(c)	int3:: int3	Wild-type::Wild-type	retained
14	LUAD	(a)/(c)	int1:: int1	Wild-type::Wild-type	retained
15	READ	N.A	Wild-type::Dup	Wild-type::Wild-type	retained
16	READ	N.A	Wild-type::Dup	Wild-type::Wild-type	retained
17	COAD	(a)/(c)	int1:: int1	3′ UTR::3′ UTR	deletion
18	GIST	(a)/(c)	int1:: int1	3′ UTR::3′ UTR	deletion
19	ESCC	(c)/(d)	int5:: int5	3′ UTR::3′ UTR	deletion
20	CCRCC	(c)/(d)	int5:: int5	3′ UTR::3′ UTR	deletion

^a^ Types of amplicons using specific PCR primers. (a)–(d) correspond to hybrids (a)–(d) in Fig. 2A.

^b^ The genome structure is in order from *CYP2A7* to *CYP2A6*. Even if an SNV was detected, if there was no copy number or structural variant, *CYP2A7*/*CYP2A6* was defined as wild-type.

^c^ Deletion: *CYP2A6* with a deletion or hybrid in both alleles; retained: normal *CYP2A6* in at least one allele.

N.A., not amplified; IGR, intergenic region; UTR, untranslated region; Dup, duplication; int, intron; SVs, structural variants.

### Association between normal CYP2A6 and mutation accumulation in tumors

**Fig. 3:**
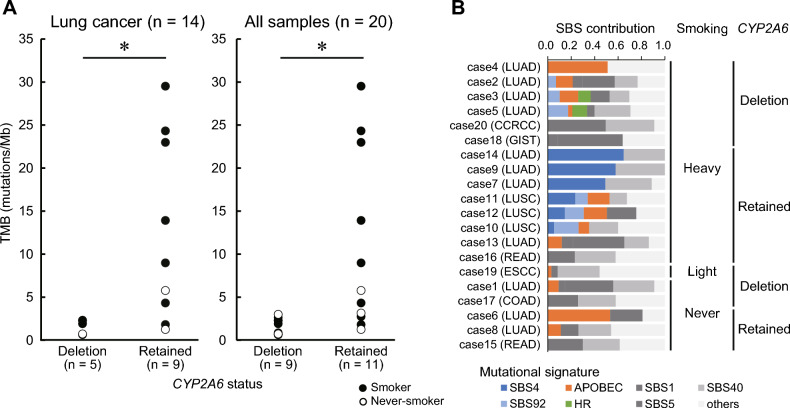
Mutation accumulation in tumors. (**A**) Tumor mutation burden (TMB) distribution in *CYP2A6* deletion and the retained types. The left panel depicts lung cancer samples, whereas the right panel shows all samples, including lung cancers. “Deletion type” (functionally deficient) denotes biallelic loss of CYP2A6 activity (e.g., CYP2A6*4 or two predicted no-function alleles including *CYP2A7/CYP2A6* hybrids). “Retained type” (functionally non-deficient) denotes ≥ 1 functional *CYP2A6* allele. *P < 0.05 (Welch’s *t*-test). (**B**) Mutational signatures of *CYP2A6* deletion- and retained-type tumors. The APOBEC signature comprises SBS2 and SBS13. Signatures with contribution rates < 0.2 in each sample, excluding smoking-related signatures (SBS4 and SBS92), were merged with others. SBS analysis was conducted using COSMIC v.3.3.

CYP2A6 is a crucial enzyme involved in nicotine metabolism, and its activity varies depending on the *CYP2A6* genotype. The accumulation of mutations due to the exposure of cells to 4-(methylnitrosamino)-1-(3-pyridyl)-1-butanone (NNK), a nicotine metabolite produced by CYP2A6, has been reported previously^17^. We investigated the effect of the presence or absence of normal germline *CYP2A6* on tumor mutational burden (TMB) (Fig. 3A). In lung cancer, TMB was significantly lower in the absence of normal *CYP2A6* (deletion type) than in the retained type, regardless of the smoking status of the patient. No tumors of the *CYP2A6* deletion type had a TMB above 10. No correlation was observed between TMB and the copy number of normal germline *CYP2A6* in the retained-type tumors (data not shown). Furthermore, the smoking-related signature, SBS4, was not detected in lung cancers with a deletion type, even in heavy smokers, but was detected in 86% (6 of 7) of lung cancers with a retained type (Fig. 3B, Supplementary Figure S3). In 75% (3 of 4) of the deletion-type lung cancers in patients with a history of smoking, we found SBS92, often observed in bladder cancer. No significant increase in TMB was observed in the retained-type tumors other than LUAD and LUSC, regardless of the smoking status, and SBS4 and SBS92 were not detected. These results suggest that lung cancers with genomically conserved *CYP2A6* accumulate somatic mutations associated with SBS4 and the accumulation increases with smoking.

## Discussion

In this study, we identified three novel *CYP2A7/CYP2A6* hybrids in Japanese patients with cancer. Using LRS, the hybrids were confirmed to switch among introns 1, 3, and 5 of *CYP2A6* and *CYP2A7*, with a high sequence similarity; additionally, the sequences were verified using Sanger sequencing. Despite the high degree of homology, LRS correctly recognized the *CYP2A7*/*CYP2A6* hybrid sequence, including the SP, and the haplotype could be evaluated. SRS detected CNVs but could not resolve gene-to-gene switching (hybrid formation) between *CYP2A6* and *CYP2A7* because short reads are difficult to map uniquely in highly homologous regions. Although the LRS used in this study relies on Illumina’s sequencing-by-synthesis with molecular barcoding to reconstruct kilobase-scale reads (mean 5–7 kb from ~ 250 ng DNA), common long-read platforms such as PacBio generate true single-molecule real-time (SMRT) reads by observing a polymerase in zero-mode waveguides, typically yielding longer read-length distributions (15–20 kb).

As *CYP2A6* and its neighboring *CYP2A7* show more than 90% sequence identity, accurately detecting the switch region between *CYP2A6* and *CYP2A7* using conventional PCR-based genotyping is difficult^18^. Langlois et al*.*^19^ reported that SV imputation using SNP arrays identified multiple hybrid alleles in *CYP2A6*. Similar to *CYP2A7/CYP2A6* hybrids, *CYP2D6* has multiple variants involving gene hybrids with the neighboring *CYP2D7*. Charnaud et al.^11^ successfully performed high-resolution typing of the complex *CYP2D6* locus using PacBio long-read amplicon sequencing and noted the importance of using LRS to obtain detailed information on critical cytochrome P450 genes, such as those in the Pharmacogene Variation (PharmVar) Consortium. Recently, NGS technologies have been developed that can call SVs in gene pairs, including pseudogenes in highly homologous regions, using SRS alone^20,21^. These methods are best suited for known genotyping and star allele/diplotype calling. We identified *CYP2A7*/*CYP2A6* hybrids that have not been reported from SNP-array–based imputation in prior studies^19^ using a combination of LRS and SRS; therefore, we believe that LRS is important for discovering unknown SVs in highly homologous regions.

Products of known *CYP2A7*/*CYP2A6* hybrids, including CYP2A6*12, CYP2A6*34, CYP2A6*47, and CYP2A6*53, are predicted to have extremely low or no CYP2A6 activity^19,22,23^. These hybrids occur in parts of Europe^5^. In the present study, the presence of hybrids and complete UTR deletions of *CYP2A6* were due to switching with the nearby gene *CYP2A7* on the same chromosome, and these switching regions were close to the previously reported CYP2A6*12 and *34 hybrid encoding products without enzyme activity. Regarding the known hybrids, these SVs were predicted to result in severely reduced or no functional activity, depending on allele composition, based on the results of PharmVar. Therefore, we classified cases in which the complete *CYP2A6* structure was maintained in one allele as ‘retained type’ and those in which *CYP2A6* function was predicted to be lost in both alleles due to deletion or SV as ‘deletion type’.

The frequency of deletion of both *CYP2A6* alleles varies among ethnic groups; it is rare in the European and American populations but more frequent in the East Asian populations, including Japan^5,10^. In populations with this homozygous deletion, the risk of developing lung cancer is low^24^. Because all participants were Japanese, the allele frequencies and biological effects of the novel *CYP2A7*/*CYP2A6* hybrids identified here may not be directly extrapolated to other populations. Validation in larger, multi-ancestry cohorts is required to assess their prevalence and potential impact across different ethnic groups. In addition, previous assessments of *CYP2A6* deletion did not include the novel hybrids reported here. If these hybrids result in loss of function of CYP2A6, the risk of lung cancer development should be re-evaluated to account for these SVs. Furthermore, the three novel CYP2A7/CYP2A6 hybrids identified here are not represented in public pharmacogenomic databases such as PharmVar. Their population frequencies and clinical penetrance therefore remain unknown, and future large-scale surveys in diverse populations will be required to establish their prevalence and biological impact.

Tobacco chemicals, such as NNK, that are metabolized by CYP2A6 may cause mutations to accumulate^17^. We previously detected complete *CYP2A6* deletion (not including the hybrids identified in the present study) in 22 of 559 patients with LUAD but not in 151 patients with LUSC, which is thought to be strongly related to smoking^8^. Therefore, we hypothesized that patients with *CYP2A6* deletion accumulate fewer smoking-derived mutations, as they are not exposed to CYP2A6 metabolites, which lead to the accumulation of tobacco-derived mutations. TMB and mutational signature analyses revealed that the deletion type of *CYP2A6* accumulated fewer smoking-related mutations than the retained type. SBS4, associated with smoking, is thought to be partly due to exposure to benzo[a]pyrene (BaP) present in tobacco according to in vitro experiments^17,25,26^. Notably, although the absence of CYP2A6 activity appears to prevent mutation accumulation, the CYP1 family (CYP1A1, CYP1A2, and CYP1B1), rather than CYP2A6, may contribute to the metabolism of BaP and its metabolites promote mutation accumulation as DNA adducts ^27–29^. These results suggest that CYP2A6 may contribute to the generation of DNA adducts derived from BaP or that it may metabolize tobacco-derived chemicals that lead to the formation of SBS4 other than BaP.

To contextualize our TMB values against larger cohorts, we benchmarked them using WES-based references. Jiao et al. reported a median TMB of 5.1 mutations/Mb in a Chinese multi-cancer cohort^30^, and analyses of the predominantly White TCGA-LUAD cohort cite a median of approximately 5.78 mutations/Mb^31^. In our cohort, the *CYP2A6* deletion type tumors were all < 2.5 mutations/Mb, which places them below these medians. Because TMB estimation can vary across studies (e.g., exome capture size and variant-calling pipelines), these external values are used for descriptive benchmarking rather than formal statistical comparison.

The present study is the first to reveal that germline SVs may contribute to suppressing mutation accumulation in tumors. However, because this study was based on preselected cases enriched for CYP2A6 structural variants from our previous studies^9,13,14^, the sample size is small and statistical power is limited. Therefore, further validation in larger, population-based cohorts will be required to generalize these findings. The loss of CYP2A6 activity due to the hybrids identified in this study remains to be demonstrated. Although the structural configuration of these hybrids suggests a likely reduction or loss of enzymatic activity, direct functional assays will be required to confirm their impact.

At present, whether *CYP2A6* structural status can function as a biomarker for therapeutic response or cancer risk stratification remains uncertain and should be addressed in future studies. Importantly, our findings suggest a link between germline structural variants and reduced mutational accumulation, which could have implications for the development of personalized preventive medicine. It also remains unclear whether similar genomic mechanisms operate in other enzymes involved in carcinogen metabolism. Nevertheless, the combined LRS/SRS strategy described here could be broadly applied to other highly homologous genomic regions, which may facilitate the discovery of additional germline structural variants with functional or clinical relevance.

Overall, combined whole-genome SRS and LRS could facilitate the detection of SVs representing unknown *CYP2A6* variants, thus elucidating the functional mechanisms of *CYP2A6* and/or lung carcinogenesis in smokers. The proposed NGS approach could help evaluate the effects of unknown germline SVs in homologous regions on tumor development. Furthermore, understanding germline SVs and mutational accumulation patterns in lung cancer may be useful for treating lung cancer with immune checkpoint inhibitors and preventing individual diseases.

## Materials and methods

### Patients

We selected patients with LUAD (*n* = 11), LUSC (*n* = 3), rectal adenocarcinoma (READ, *n* = 2), colon adenocarcinoma (COAD, *n* = 1), gastrointestinal stromal tumor (GIST, *n* = 1), esophageal squamous cell carcinoma (ESCC, *n* = 1), and clear cell renal cell carcinoma (CCRCC, *n* = 1) who potentially exhibited genomic CNVs in *CYP2A6* based on preliminary SRS analysis or DME gene panel sequencing, as reported previously^8^. The patients were treated at the Shizuoka Cancer Center Hospital, and tumor and blood samples were obtained from those who had undergone surgery. Samples with tumors > 100 mg were analyzed. The study protocols (named Project HOPE) were approved by the institutional review board of the Shizuoka Cancer Centre (authorization numbers 25–33). In this study, pathogenic germline alterations were predicted in blood specimens. Appropriate informed consent, including the possibility of secondary findings such as those found in blood-based constitutional analysis, was obtained from all the patients with the approval of the ethics review board. Human studies followed the ethical guidelines for clinical application outlined in the Declaration of Helsinki.

### Whole-genome SRS

Whole-genome SRS was conducted on the Illumina NovaSeq 6000 sequencing system (Illumina Inc., San Diego, CA, USA). Libraries were prepared from 1 μg of DNA extracted from blood samples (7 × 2 ml) using the TruSeq DNA PCR-Free High Throughput Library Prep Kit (Illumina) following the manufacturer’s instructions. Germline variants, including single-nucleotide variants (SNVs), and insertions and deletions (indels) were identified using the DRAGEN Small Variant Caller and Structural Variant Caller (Illumina). SVs and CNVs were identified using the DRAGEN Structural Variant Caller and Copy Number Variant Pipeline (Illumina), respectively. Variants were annotated with consequences using the Ensembl Variant Effect Predictor (v.104)^32^. The functional consequences of the variant calls were assessed using an in-house annotation pipeline^13,14^. Mutational signature analysis was performed by using MutationalPatterns v3.0^33^. The profile was decomposed into an optimal combination of single-base substitution (SBS) in COSMIC (v.3.3), and signatures described as artifacts were excluded from the analysis.

DNA was extracted from the tumor samples of the same patients from whom whole blood was collected to calculate the tumor mutation burden (TMB) and mutational signature. Tissue DNA and blood DNA were extracted using the QIAamp DNA Mini Kit (QIAGEN, Hilden, Germany) and QIAamp DNA Blood Kit (QIAGEN), respectively. DNA was quantified using a Nanodrop (Thermo Fisher Scientific, Wilmington, DE, USA) and a Qubit 2.0 Fluorometer (Thermo Fisher Scientific). NGS was performed in accordance with a previously reported protocol^13^, and variant calls of somatic mutations were assessed using the blood as the matched control. The TMB and mutational signature were calculated from somatic mutations in the exon and whole-genome regions, respectively.

### Whole-genome LRS

Whole‑genome long‑read sequencing (LRS) was performed on an Illumina NovaSeq 6000 sequencing system. Libraries were prepared from 250 ng of genomic DNA using the Illumina Complete Long Read Prep, Human Kit (Illumina) according to the manufacturer’s instructions. Combined LRS and short‑read sequencing (SRS) data were analyzed on the Illumina Complete Long Read Whole Genome Sequencing platform. LRS data were processed with the DRAGEN Illumina Complete Long Reads Apps on the BaseSpace Sequence Hub. In this workflow, LRS reads were analyzed as follows: (1) marked sites were identified in each read based on the presence of expected landmarks; (2) pairs of reads sharing the same landmarks were detected; (3) groups of reads were clustered; (4) each cluster was assembled into a single long read; (5) marks were removed from the assembled long reads; and (6) the assembled long reads were aligned to the human reference genome using minimap2.

In the *CYP2A6*–*CYP2A7* paralogous region, switch positions were determined as follows. We first examined whether SVs were detected across the *CYP2A6*–*CYP2A7* interval based on LRS data, then evaluated copy number across the same region using SRS depth. By integrating SV calls from LRS with copy number profiles from SRS, we inferred allele‑specific genomic structures: regions with copy number 0 were interpreted as homozygous deletions, whereas regions with copy number 1 were considered heterozygous alterations. On this basis, we identified a candidate *CYP2A7::CYP2A6* hybrid. To validate this structure, we amplified a 2.5–6 kb PCR product spanning the putative junction and performed primer-walking Sanger sequencing to define the switch position, which was assigned to the sequence position where *CYP2A6* and *CYP2A7* show divergence. Targeted amplicon‑based WES was also performed, and no reads were obtained from exon‑targeted amplicons when one primers was located within the deleted region. This apparent absence of exon coverage reflects loss of the primer‑binding site rather than true exon deletion, and was used as an additional reference for identifying the bridging site.

### PCR

PCR was performed using PrimeSTAR GXL DNA Polymerase (TAKARA BIO, Shiga, Japan). The reverse primer was used commonly and the sequence was 5′-GGATTCCTCTCCCTTGGAAT-3′. The forward primer sequences were designed: 5′-GGGTATTGGACATCCATCCT-3′, 5′-GTCCAGTTTTCTTGGCATTG-3′, and 5′-TAGAAAGCTTCTAATGTGGGTG-3′ for PCR1, PCR2, and PCR3, respectively. The size of the PCR amplicon was confirmed by electrophoresis using a TapeStation (Agilent Technologies, Santa Clara, CA, USA). This device is a fully automated electrophoresis device that uses gel-filled electrophoresis cartridges.

### Statistical analysis

TMB was compared using Welch’s *t*-test. The results were considered significant at *p* < 0.05.

## Supplementary Information

Supplementary Information.

## Data Availability

The whole-genome SRS data generated herein cannot be shared publicly due to the inclusion of germline alteration sequences classified as personal identifiers in Japan. Additionally, the whole-genome LRS data in this article were provided by Illumina after obtaining relevant permissions. These data will be shared upon request to the corresponding author with permission from Illumina. The dataset of *CYP2A7* / *CYP2A6* hybrids generated and analyzed during in the current study are available in the NBDC Human Database repository (https://humandbs.dbcls.jp; temporary accession ID, hum0127.v4).

## References

[1] Ariyoshi, N. et al. Genetic polymorphism of CYP2A6 gene and tobacco-induced lung cancer risk in male smokers. *Cancer Epidemiol. Biomarkers Prev.* **11**, 890–894 (2002). 12223434

[2] Fujieda, M. et al. Evaluation of CYP2A6 genetic polymorphisms as determinants of smoking behavior and tobacco-related lung cancer risk in male Japanese smokers. *Carcinogenesis* **25**, 2451–2458 (2004). 15308589 10.1093/carcin/bgh258

[3] Patel, Y. M. et al. Novel association of genetic markers affecting CYP2A6 activity and lung cancer risk. *Cancer Res.* **76**, 5768–5776 (2016). 27488534 10.1158/0008-5472.CAN-16-0446 PMC5050097

[4] Murphy, S. E. Biochemistry of nicotine metabolism and its relevance to lung cancer. *J. Biol. Chem.* **296**, 100722 (2021). 33932402 10.1016/j.jbc.2021.100722 PMC8167289

[5] Langlois, A. W. R. et al. PharmVar GeneFocus: CYP2A6. *Clin. Pharmacol. Ther.* **116**, 948–962 (2024). 39051767 10.1002/cpt.3387 PMC11452280

[6] Liu, T., Xie, C. B., Ma, W. J. & Chen, W. Q. Association between CYP2A6 genetic polymorphisms and lung cancer: a meta-analysis of case-control studies. *Environ. Mol. Mutagen.* **54**, 133–140 (2013). 23203414 10.1002/em.21751

[7] Tremmel, R. et al. Copy number variation profiling in pharmacogenes using panel-based exome resequencing and correlation to human liver expression. *Hum. Genet.* **139**, 137–149 (2020). 31786673 10.1007/s00439-019-02093-7

[8] Ohnami, S. et al. Whole exome sequencing detects variants of genes that mediate response to anticancer drugs. *J. Toxicol. Sci.* **42**, 137–144 (2017). 28321040 10.2131/jts.42.137

[9] Ohnami, S. et al. Comparison of genetic susceptibility to lung adenocarcinoma and squamous cell carcinoma in Japanese patients using a novel panel for cancer-related drug-metabolizing enzyme genes. *Sci. Rep.* **12**, 17928 (2022). 36289279 10.1038/s41598-022-22914-6 PMC9606290

[10] Zhou, Y., Ingelman-Sundberg, M. & Lauschke, V. M. Worldwide distribution of cytochrome P450 alleles: A meta-analysis of population-scale sequencing projects. *Clin. Pharmacol. Ther.* **102**, 688–700 (2017). 28378927 10.1002/cpt.690 PMC5600063

[11] Charnaud, S. et al. PacBio long-read amplicon sequencing enables scalable high-resolution population allele typing of the complex CYP2D6 locus. *Commun. Biol.* **5**, 168 (2022). 35217695 10.1038/s42003-022-03102-8 PMC8881578

[12] Sanford Kobayashi, E. et al. Approaches to long-read sequencing in a clinical setting to improve diagnostic rate. *Sci. Rep.* **12**, 16945 (2022). 36210382 10.1038/s41598-022-20113-x PMC9548499

[13] Nagashima, T. et al. Japanese version of the cancer genome Atlas, JCGA, established using fresh frozen tumors obtained from 5143 cancer patients. *Cancer Sci.* **111**, 687–699 (2020). 31863614 10.1111/cas.14290 PMC7004528

[14] Nagashima, T. et al. Evaluation of whole genome sequencing utility in identifying driver alterations in cancer genome. *Sci. Rep.* **14**, 23898 (2024). 39396060 10.1038/s41598-024-74272-0 PMC11470963

[15] Jia, H. et al. Low-input PacBio sequencing generates high-quality individual fly genomes and characterizes mutational processes. *Nat. Commun.* **15**, 5644 (2024). 38969648 10.1038/s41467-024-49992-6 PMC11226609

[16] Cleal, K. & Baird, D. M. Dysgu: efficient structural variant calling using short or long reads. *Nucleic Acids Res.* **50**, e53 (2022). 35100420 10.1093/nar/gkac039 PMC9122538

[17] Mingard, C. et al. Dissection of cancer mutational signatures with individual components of cigarette smoking. *Chem. Res. Toxicol.* **36**, 714–723 (2023). 36976926 10.1021/acs.chemrestox.3c00021 PMC10114081

[18] Langlois, A. W. R., Pouget, J. G., Knight, J., Chenoweth, M. J. & Tyndale, R. F. Associating CYP2A6 structural variants with ovarian and lung cancer risk in the UK Biobank: Replication and extension. *Eur. J. Hum Genet.* **32**, 357–360 (2024). 38097766 10.1038/s41431-023-01518-2 PMC10923790

[19] Langlois, A. W. R. et al. Genotyping, characterization, and imputation of known and novel CYP2A6 structural variants using SNP array data. *J. Hum. Genet.* **68**, 533–541 (2023). 37059825 10.1038/s10038-023-01148-y

[20] Chen, X. et al. Spinal muscular atrophy diagnosis and carrier screening from genome sequencing data. *Genet. Med.* **22**, 945–953 (2020). 32066871 10.1038/s41436-020-0754-0 PMC7200598

[21] Chen, X. et al. Cyrius: accurate CYP2D6 genotyping using whole-genome sequencing data. *Pharmacogenomics J.* **21**, 251–261 (2021). 33462347 10.1038/s41397-020-00205-5 PMC7997805

[22] Oscarson, M. et al. Characterization of a novel CYP2A7/CYP2A6 hybrid allele (CYP2A6*12) that causes reduced CYP2A6 activity. *Hum. Mutat.* **20**, 275–283 (2002). 12325023 10.1002/humu.10126

[23] Oscarson, M. et al. Identification and characterisation of novel polymorphisms in the CYP2A locus: implications for nicotine metabolism. *FEBS Lett.* **460**, 321–327 (1999). 10544257 10.1016/s0014-5793(99)01364-2

[24] Johani, F. H., Majid, M. S. A., Azme, M. H. & Nawi, A. M. Cytochrome P450 2A6 whole-gene deletion (CYP2A6*4) polymorphism reduces risk of lung cancer: A meta-analysis. *Tob. Induc. Dis.* **18**, 50 (2020). 32547353 10.18332/tid/122465 PMC7291960

[25] Olivier, M. et al. Modelling mutational landscapes of human cancers in vitro. *Sci. Rep.* **4**, 4482 (2014). 24670820 10.1038/srep04482 PMC5259794

[26] Kucab, J. E. et al. A compendium of mutational signatures of environmental agents. *Cell* **177**, 821-836 e816 (2019). 30982602 10.1016/j.cell.2019.03.001 PMC6506336

[27] Chung, J. Y. et al. Cellular defense mechanisms against benzo[a]pyrene in testicular Leydig cells: implications of p53, aryl-hydrocarbon receptor, and cytochrome P450 1A1 status. *Endocrinology* **148**, 6134–6144 (2007). 17884947 10.1210/en.2007-0006

[28] Shiizaki, K., Kawanishi, M. & Yagi, T. Modulation of benzo[a]pyrene-DNA adduct formation by CYP1 inducer and inhibitor. *Genes Environ.* **39**, 14 (2017). 28405246 10.1186/s41021-017-0076-x PMC5385587

[29] Barnes, J. L., Zubair, M., John, K., Poirier, M. C. & Martin, F. L. Carcinogens and DNA damage. *Biochem Soc Trans.* **46**, 1213–1224 (2018). 30287511 10.1042/BST20180519 PMC6195640

[30] Jiao, X. D. et al. Tumor mutation burden in Chinese cancer patients and the underlying driving pathways of high tumor mutation burden across different cancer types. *Ann Transl Med.* **8**, 860 (2020). 32793704 10.21037/atm-20-3807 PMC7396744

[31] Cancer Genome Atlas Research, N. Comprehensive molecular profiling of lung adenocarcinoma. *Nature*. **511**, 543–550 (2014). 10.1038/nature13385 PMC4231481 25079552

[32] McLaren, W. et al. The ensembl variant effect predictor. *Genome Biol.* **17**, 122 (2016). 27268795 10.1186/s13059-016-0974-4 PMC4893825

[33] Manders, F. et al. MutationalPatterns: The one stop shop for the analysis of mutational processes. *BMC Genom.* **23**, 134 (2022). 10.1186/s12864-022-08357-3 PMC8845394 35168570

