# Supplementary material for: Novel CYP2A7/CYP2A6 germline hybrids associated with mutation burden in lung cancer revealed by whole-genome long-read sequencing
Source: Sci Rep. 2025 Nov 21;15:44989. doi: 10.1038/s41598-025-29542-w (PMC12749657; doi:10.1038/s41598-025-29542-w)
Supplement: Supplementary file 1 — Supplementary Information. [file 41598_2025_29542_MOESM1_ESM.pdf]

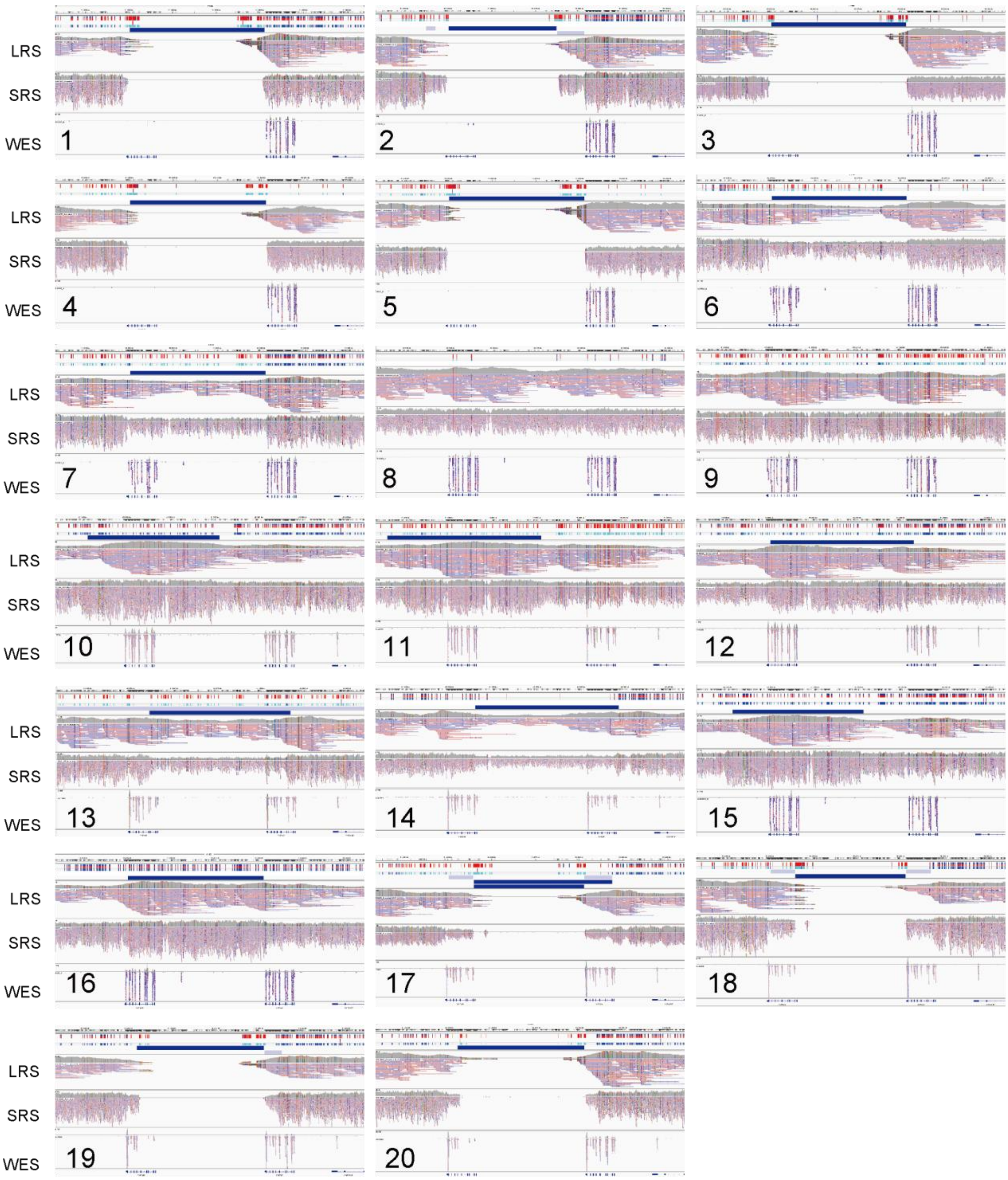

Supplementary Figure S1. Integrative genomics viewer (IGV) images of all samples. Mapped long-read sequencing (LRS), short-read sequencing (SRS), and whole-exome sequencing (WES) reads for the *CYP2A6* and *CYP2A7* regions are shown. Detected variants are shown at the top of each panel. Dark blue bars indicate regions with SVs detected in LRS. The number at the bottom left is the sample number.

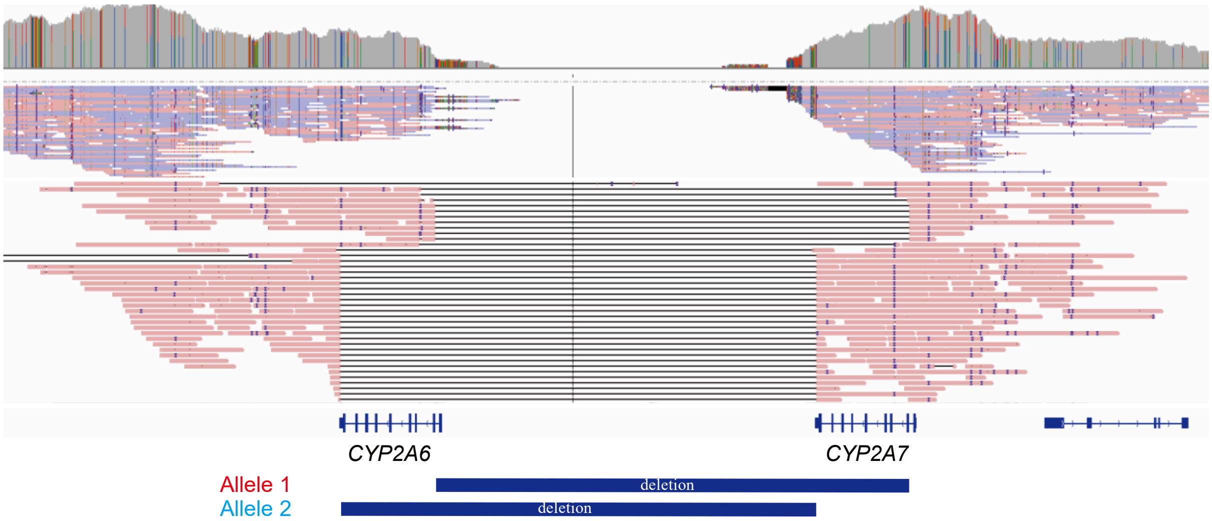

Supplementary Figure S2. BLAT re-alignment of long-read sequencing data in a representative case (Case No. 17) with a novel *CYP2A7*/*CYP2A6* hybrid allele. Integrative Genomics Viewer (IGV) snapshot showing long-read sequencing (LRS) data across the *CYP2A6–CYP2A7* locus. BLAT re-alignment of LRS mapped them to positions corresponding to each allele with a deletion between *CYP2A6* and *CYP2A7*, thereby resolving the ambiguous mappings observed in IGV.

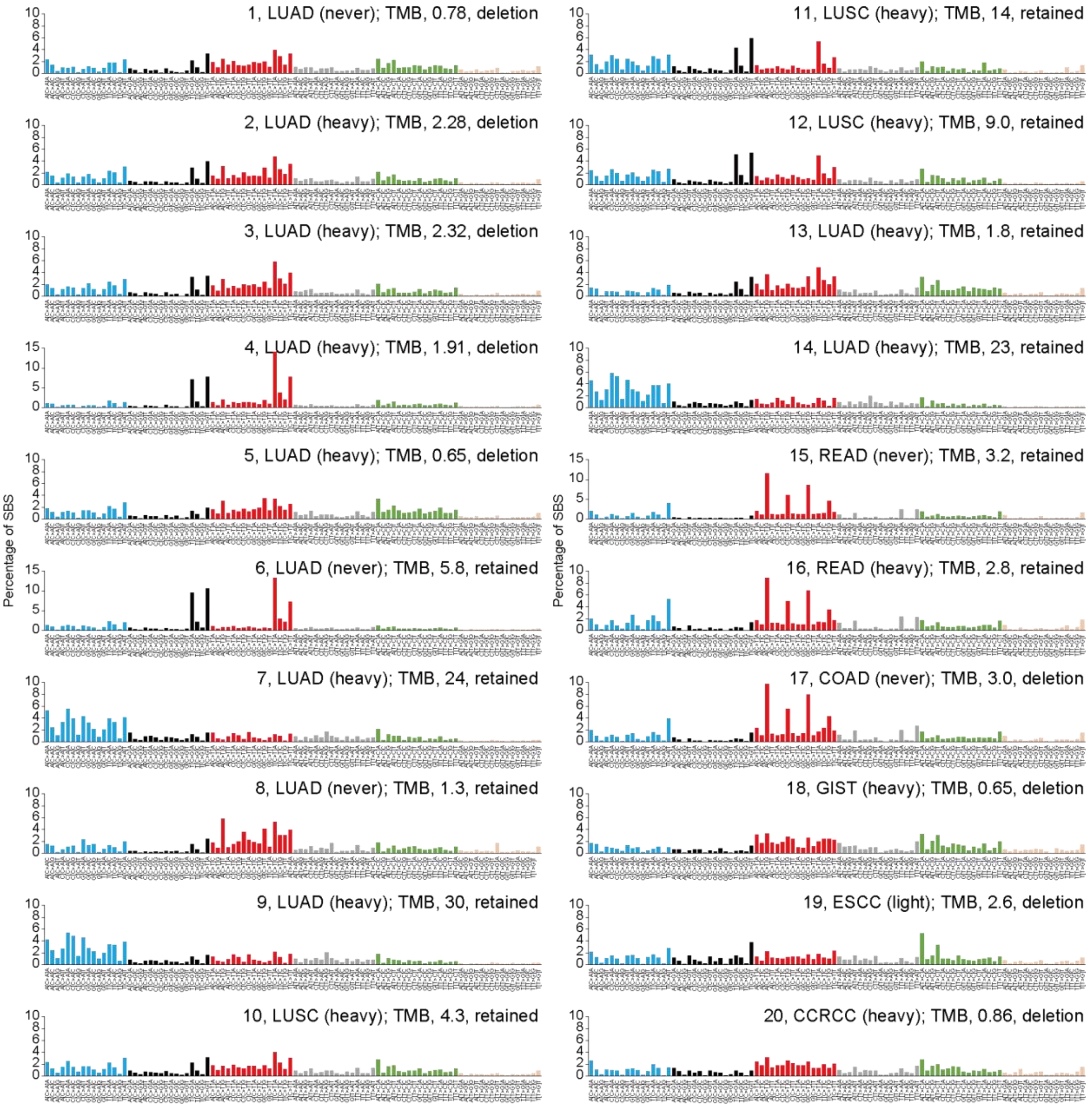

Supplementary Figure S3. Tumor mutation profile. The profile represents 96 conventional mutation patterns based on somatic mutations detected using WGS. These patterns are classified according to six substitution subtypes and the bases on either sides.

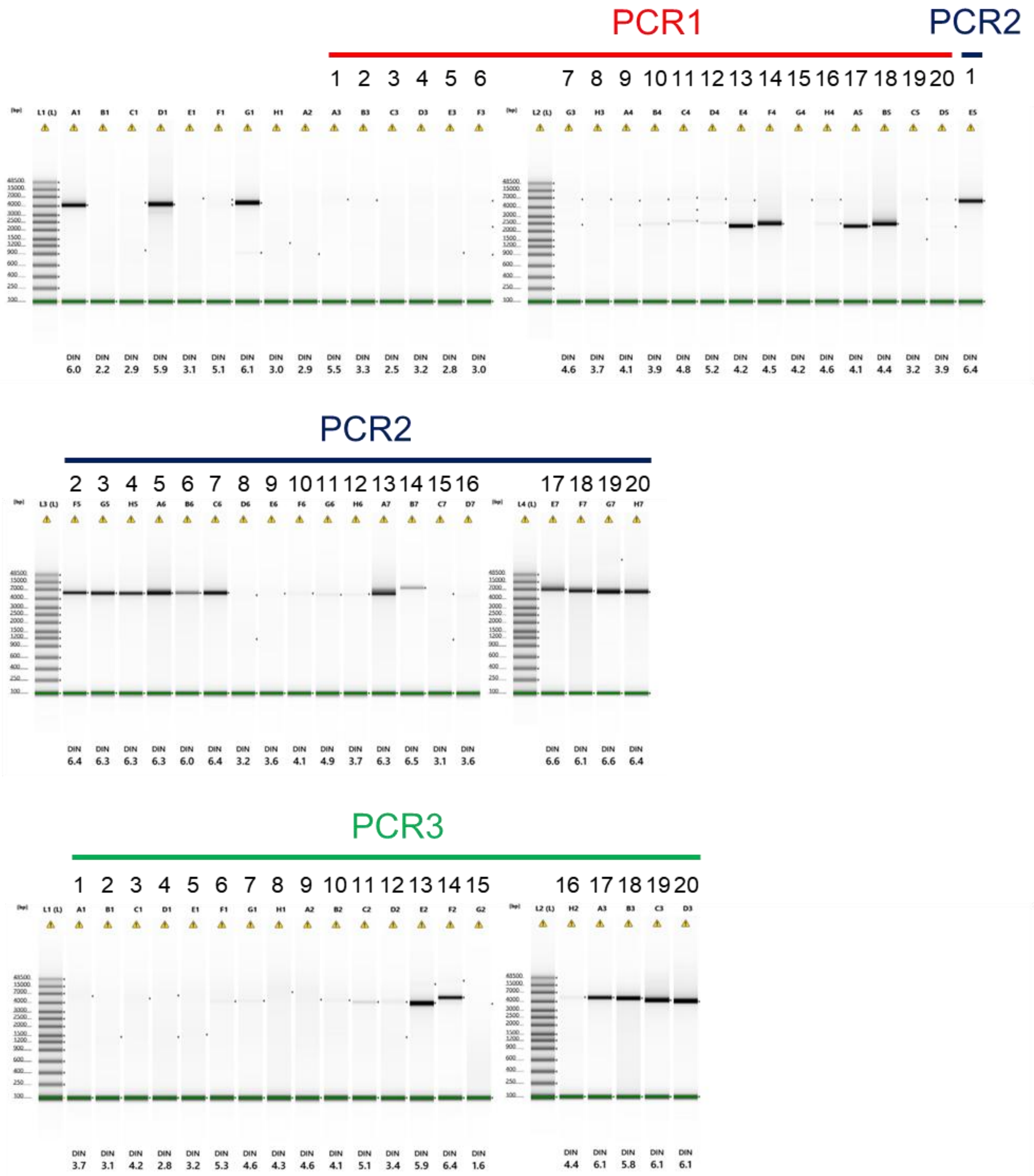

Supplementary Figure S4. Raw electrophoresis data output from the TapeStation. Case numbers and PCR1–3 correspond to Figure 2.

Supplementary Table S1. Structural variants (SVs) and switch positions estimated in each allele.

Case No.	Allele	Genomic structure in *CYP2A7*/*CYP2A6* hybrid[a]	Locus of switches		Smoking status	Tumor types	Star allele (hybrid)
			Breakpoint position[b]	SV types of *CYP2A6*			
1	Allele 1	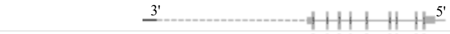	chr19:41349396–41381295	Del	Never	LUAD	*4 (c)
	Allele 2	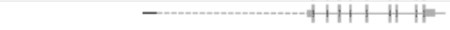	chr19:41349322–41381206	Del			*4 (c)
2	Allele 1	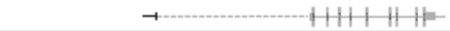	chr19:41349596–41381495	Del	Heavy	LUAD	*4 (c)
	Allele 2	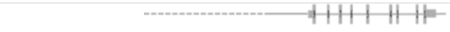	chr19:41344999–41374912*	Del			*4 (c)
3	Allele 1	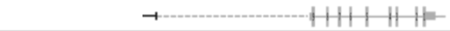	chr19:41349596–41381495	Del	Heavy	LUAD	*4 (c)
	Allele 2	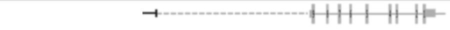	chr19:41349596–41381495	Del			*4 (c)
4	Allele 1	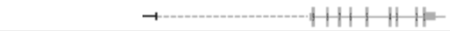	chr19:41349596–41381495	Del	Heavy	LUAD	*4 (c)
	Allele 2	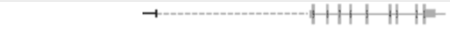	chr19:41349596–41381495	Del			*4 (c)
5	Allele 1	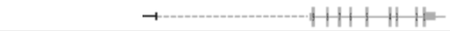	chr19:41349596–41381495	Del	Heavy	LUAD	*4 (c)
	Allele 2	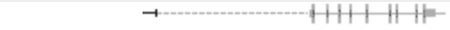	chr19:41349596–41381495	Del			*4 (c)
6	Allele 1	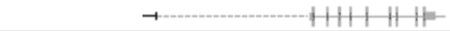	chr19:41349596–41381495	Del	Never	LUAD	*4 (c)
	Allele 2	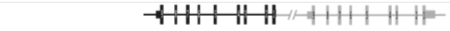	wild–type				*1
7	Allele 1	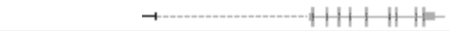	chr19:41349596–41381495	Del	Heavy	LUAD	*4 (c)
	Allele 2	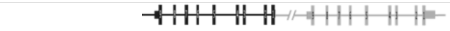	wild–type				*1
8	Allele 1	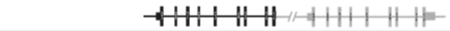	wild–type		Never	LUAD	*1
	Allele 2	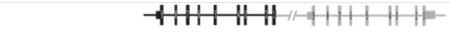	wild–type			(control)	*1
9	Allele 1	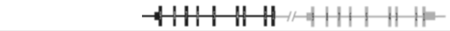	wild–type		Heavy	LUAD	*1
	Allele 2	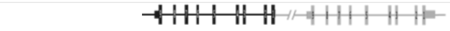	wild–type			(control)	*1
10	Allele 1	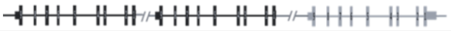	chr19:41341137–41371167*	Dup	Heavy	LUSC	*1x2
	Allele 2	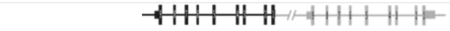	wild–type				*1
11	Allele 1	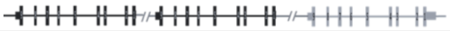	chr19:41341137–41371167*	Dup	Heavy	LUSC	*1x2
	Allele 2	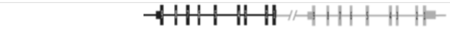	wild–type				*1
12	Allele 1	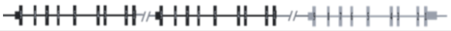	chr19:41350217–41383124*	Dup	Heavy	LUSC	*1x2
	Allele 2	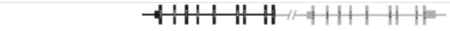	wild–type				*1
13	Allele 1	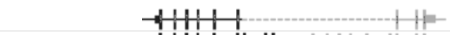	chr19:41354500–41386367	hybrids with *CYP2A7*	Heavy	LUAD	novel (b)
	Allele 2	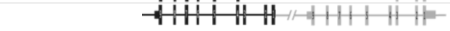	wild–type				*1
14	Allele 1	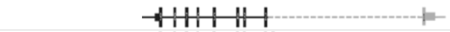	chr19:41355896–41387669	hybrids with *CYP2A7*	Heavy	LUAD	novel (a)
	Allele 2	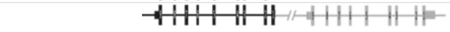	wild–type				*1
15	Allele 1	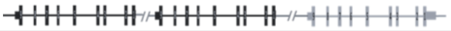	chr19:41341137–41371167*	Dup	Never	READ	*1x2
	Allele 2	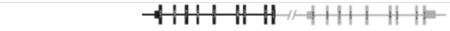	wild–type				*1
16	Allele 1	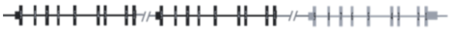	chr19:41350217–41381210*	Dup	Heavy	READ	*1x2
	Allele 2	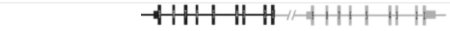	wild–type				*1
17	Allele 1	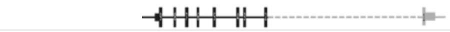	chr19:41355896–41387669	hybrids with *CYP2A7*	Never	COAD	novel (a)
	Allele 2	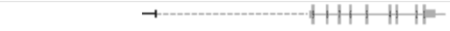	chr19:41349596–41381495	Del			*4
18	Allele 1	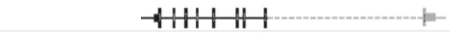	chr19:41355896–41387669	hybrids with *CYP2A7*	Heavy	GIST	novel (a)
	Allele 2	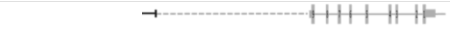	chr19:41349596–41381495	Del			*4 (c)
19	Allele 1	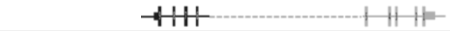	chr19:41352499–41384385	hybrids with *CYP2A7*	Light	ESCC	novel (d)
	Allele 2	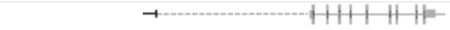	chr19:41349596–41381495	Del			*4 (c)
20	Allele 1	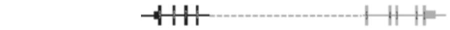	chr19:41352499–41384385	hybrids with *CYP2A7*	Heavy	CCRCC	novel (d)
	Allele 2	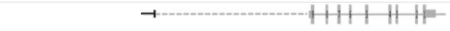	chr19:41349596–41381495	Del			*4 (c)

[a] The dotted line represents the deletion region. The black and grey boxes indicate the *CYP2A6* and *CYP2A7* exons, respectively. The consecutive *CYP2A6* indicates a duplication.

[b] Locus indicates estimated switch positions for *CYP2A6* and *CYP2A7*.

* The switch position could not be verified in cases with complete deletion of the intergenic region downstream of *CYP2A6* or a duplication of *CYP2A6*, but the breakpoint was confirmed

by long-read sequencing.

Star allele (hybrid) indicates PharmVar star-allele designations where available; “novel (a/b/d)” denotes newly identified hybrids without existing PharmVar star designations. (a–d) follow Figure 2.

DEL, deletion; DUP, duplication; LUAD, Lung adenocarcinoma; LUSC, Lung squamous cell carcinoma; READ, Rectal adenocarcinoma; COAD, Colon adenocarcinoma; GIST, Gastrointestinal stromal tumor; ESCC, Esophageal squamous cell carcinoma; CCRCC, Clear cell renal cell carcinoma.
